# A method to measure renal inner medullary perfusion using MR renography

**DOI:** 10.1007/s10334-025-01225-7

**Published:** 2025-02-15

**Authors:** A. de Boer, K. Sharma, B. Alhummiany, S. P. Sourbron

**Affiliations:** 1 Imaging Division, University Medical Center Utrecht, Utrecht University, Utrecht, The Netherlands; 2 Antaros Medical AB, Mölndal, Sweden; 3 Division of Clinical Medicine, University of Sheffield, Sheffield, UK

**Keywords:** Kidney medulla, Perfusion, Magnetic resonance imaging

## Abstract

**Objective:**

In the kidney, the medulla is most susceptible to damage in case of hampered perfusion or oxygenation. Due to separate regulation of cortical and medullary perfusion, measurement of both is crucial to improve the understanding of renal pathophysiology. We aim to develop and evaluate a physiologically accurate model to measure renal inner medullary (F_med_) and cortical perfusion (F_cor_) separately.

**Materials and methods:**

We developed a 7-compartment model of renal perfusion and used an iterated approach to fit 10 free parameters. Model stability and accuracy were tested on both patient data and simulations. Cortical perfusion and F_T_ (tubular flow or glomerular filtration rate per unit of tissue volume) were compared to a conventional 2-compartment filtration model.

**Results:**

Average (standard deviation) F_med_ was 37(23)mL/100 mL/min. Fitting stability as expressed by the median (interquartile range) coefficient of variation between fits was 0.0(0.0–5.8)%, with outliers up to 81%. In simulations, F_med_ was underestimated by around 8%. Intra-class correlation coefficients for F_cor_ and F_T_ as measured with the 2- and 7- compartment model were 0.87 and 0.63, respectively.

**Discussion:**

We developed a pharmacokinetic model closely following renal physiology. Although the results were vulnerable for overfitting, relatively stable results could be obtained even for F_med_.

**Supplementary Information:**

The online version contains supplementary material available at 10.1007/s10334-025-01225-7.

## Introduction

The renal medulla functions at the border of hypoxia, even under physiological conditions. It receives only 10 (inner medulla) to 40% (outer medulla) of renal blood flow. Furthermore, due to countercurrent exchange, oxygen can diffuse freely from arterial to venous vasa recta, further diminishing oxygen supply [1]. While nephrons in the inner medulla are adapted to these hypoxic conditions and largely rely on anaerobic respiration, the high oxygen demand of the outer medulla necessitates aerobic metabolism, leaving this region susceptible to hypoxic damage [1]. Strict renal autoregulation keeps renal (medullary) oxygenation within limits by tuning both oxygen supply and demand. Notably, increased cortical perfusion generally leads to an increase in glomerular filtration rate (GFR), resulting in more solutes being delivered to the medullary tubules. This increases tubular transport and consequently increases oxygen consumption in the medulla, which contributes to medullary hypoxia. Counterintuitively, an increase in renal perfusion and oxygen supply, benefitting cortical oxygenation, can therefore contribute to medullary hypoxia. Cortical and medullary perfusion and oxygenation therefore are regulated separately [1]. To obtain detailed insights into the role of perfusion and oxygenation in renal disease, cortical and medullary regions should also be measured separately.

Renal regional hypoxia has been extensively studied by BOLD MRI, sometimes yielding contradictory results [2]. One major drawback of BOLD MRI is its reliance on the content of deoxyhemoglobin, which leads to confounding by blood volume and perfusion [3]. For correct interpretation of BOLD MRI data, knowledge of underlying changes in blood volume and perfusion is crucial. Furthermore, although perfusion is evidently not the sole determinant of renal oxygenation, impaired perfusion is a major factor in the development of renal hypoxia. Due to the separate regulation of cortical and medullary perfusion, in vivo measurement of both renal cortical and medullary perfusion is crucial to improve understanding of the pathophysiology of renal disease.

Several techniques exist to measure total or cortical perfusion in vivo, including para-aminohippurate clearance, arterial spin labeling (ASL) and dynamic contrast-enhanced (DCE) MRI, all with their own limitations. However, currently, there are no available non-invasive techniques for reliable measurement of medullary perfusion. ASL MRI relies on an endogenous tracer which decays within a few seconds, before it is able to travel from the labeling location (the aorta) to the peritubular (medullary) vasculature. ASL measurements of medullary perfusion are therefore deemed unreliable in a recent recommendation paper [4]. DCE analysis relies on pharmacokinetic modeling of the contrast enhancement curve. The most commonly used models do not discriminate between cortex and medulla and therefore cannot measure cortical and medullary perfusion separately [5–8], while another model does discriminate between cortex and medulla but does not allow for measurement of medullary perfusion [9].

The aim of the current study is to develop and evaluate a method for measuring renal (inner) medullary perfusion in particular, by means of a physiologically accurate model. The model will be tested for precision and accuracy using both simulated and real data. Furthermore, values obtained using the model will be compared to values obtained using a conventional two-compartment model [5] and literature values.

## Methods

### Theory

**Fig. 1:**
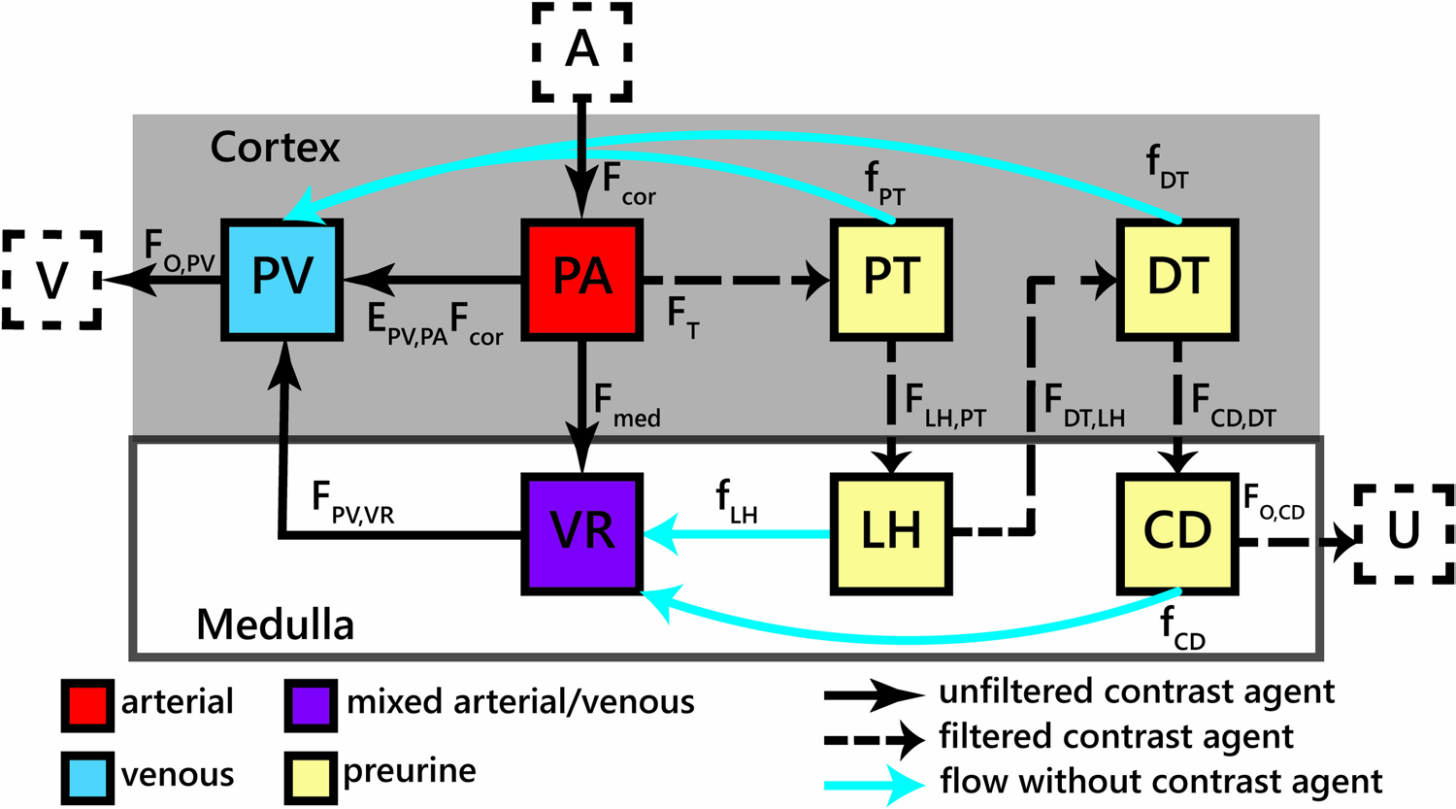
Schematic representation of the seven-compartment model. Flows with tracer are denoted F_X,Y_ where the flow is from compartment X to compartment Y or by an extraction fraction times F_cor_ (the arterial flow into the cortex). Resorption or tracer free flows are expressed as fractions f_X_ of F_T_ (E_FF_F_cor_), with X the compartment from which resorption occurs. Note that the arterial plasma compartment is not strictly arterial since it also includes glomerular and peritubular capillaries. *A* renal arteries, *PA* arterial plasma compartment, *PV* venous plasma compartment, *V* renal veins, *VR* vasa recta compartment, *PT* proximal tubules compartment, *LH* loop of Henle compartment, *DT* distal tubules compartment, *CD* collecting duct compartment, *U* urine/renal calyces

In view of our aim to measure medullary perfusion, i.e., blood flow from cortex to medulla, we identify the simplest model that can correctly describe the exchange of contrast agent between cortex and medulla (Fig. 1). A contrast agent molecule can follow essentially two trajectories between these two regions:

1. molecules that are filtered out of the plasma compartment (PA, cortex) enter the proximal tubules (PT, cortex) then the loop of Henle (LH, medulla), the distal tubules (DT, cortex) and the collecting ducts (CD, medulla) before being evacuated as urine (U);
2. molecules that are not filtered out enter the vasa recta (VR, medulla) then the venous plasma (PV, cortex) before being evacuated as venous blood (V), or else they stay in the cortex and go straight to the venous plasma (PV, cortex).

We end up with a 7-compartment model (Fig. 1) with 3 plasma compartments (PA, VR, PV) and 4 tubular compartments (PT, LH, DT, CD). The model is fully defined by 10 free parameters, summarized in Table 1. They are the mean transit times (MTT) of each of the 7 compartments, the cortical perfusion F_cor_, the medullary extraction fraction E_med_, and the glomerular extraction fraction E_FF_. The target parameter medullary perfusion F_med_ can be derived as F_med_ = E_med_(1-E_FF_)F_cor_.

**Table 1:** A list of primary and derived model parameters with units and their description. MTT: mean transit time.

Parameter	Unit	Description	Bounds
MTT_PA_	S	MTT of arterial plasma compartment	0–15
MTT_PV_	S	MTT of venous plasma compartment	1–20
MTT_VR_	S	MTT of vasa recta compartment	50–200
MTT_PT_	S	MTT of proximal tubules compartment	1–100
MTT_LH_	s	MTT of loop of Henle compartment	1–100
MTT_DT_	s	MTT of distal tubules compartment	1–100
MTT_CD_	s	MTT of collecting duct compartment	1–100
F_cor_	mL/100mL/min	Cortical perfusion	0–1000
E_med_	–	Medullary flow fraction	0–1
E_FF_	–	Glomerular extraction fraction	0–1
F_med_	mL/100mL/min	Medullary perfusion	Derived
F_T_	mL/100mL/min	Tubular flow (or GFR per 100mL tissue)	Derived

Parameters in bold are the 10 free primary parameters and are also provided with their bounds

If we write MTT_X_ for the MTT of a compartment X, then we can define the propagator H_X_ and residue functions R_X_ of the compartment as follows:

$$R_{X} = e^{ - \frac{t}{MTT_{X}}} H_{X} = \frac{e^{ - \frac{t}{MTT_{X}}}}{MTT_{X}}$$

With these definitions, the solutions of the model equations can be written out compactly by following the trajectory of the tracer flux from the arterial inlet to the compartment. For a given arterial concentration C_A_(t), the concentration in each of the 7 compartments is given by ($*$ denotes convolution):

1 $$C_{PA} \left( t \right) = \frac{1}{{V_{PA} }}R_{PA} *F_{cor} C_{A} \left( t \right)$$

2 $$C_{PV} \left( t \right) = \frac{1}{{V_{PV} }}\left[ {R_{PV} *H_{PA} *\left( {1 - E_{med} } \right)\left( {1 - E_{FF} } \right)F_{cor} C_{A} \left( t \right) + R_{PV} *H_{VR} *H_{PA} *E_{med} \left( {1 - E_{FF} } \right)F_{cor} C_{A} \left( t \right)} \right]$$

3 $$C_{VR} \left( t \right) = \frac{1}{{V_{VR} }}R_{VR} *H_{PA} *E_{med} \left( {1 - E_{FF} } \right)F_{cor} C_{A} \left( t \right)$$

4 $$C_{PT} \left( t \right) = \frac{1}{{V_{PT} }}R_{PT} *H_{PA} *E_{FF} F_{cor} C_{A} \left( t \right)$$

5 $$C_{LH} \left( t \right) = \frac{1}{{V_{LH} }}R_{LH} *H_{PT} *H_{PA} *E_{FF} F_{cor} C_{A} \left( t \right)$$

6 $$C_{CD} \left( t \right) = \frac{1}{{V_{CD} }}R_{CD} *H_{DT} *H_{LH} *H_{PT} *H_{PA} *E_{FF} F_{cor} C_{A} \left( t \right)$$

7 $$C_{DT} \left( t \right) = \frac{1}{{V_{DT} }}R_{DT} *H_{LH} *H_{PT} *H_{PA} *E_{FF} F_{cor} C_{A} \left( t \right)$$

It is unlikely that a model of this complexity can be identified based only on a measured curve over the entire kidney parenchyma. Instead, we follow a strategy introduced by Baumann et al. [10] of collecting concentrations in the cortex and medulla separately. Since medullary perfusion quantifies the exchange between these two regions, we hypothesized that this would produce a well-defined inverse problem. The cortex consists of the arterial plasma, venous plasma, proximal tubules and distal tubules:

8 $$C_{cor} \left( t \right) = V_{PV}C_{PV} \left( t \right) + V_{PA}C_{PA} \left( t \right) + V_{PT}C_{PT} \left( t \right) + V_{DT}C_{DT} \left( t \right)$$

The (fractional) volumina correct for the relative contribution of each compartment and cancel out in the final equation. The medulla consists of the vasa recta, loop of Henle, and collecting ducts:

9 $$C_{med} \left( t \right) = V_{VR}C_{VR} \left( t \right) + V_{LH}C_{LH} \left( t \right) + V_{CD}C_{CD} \left( t \right)$$

### Patient data collection

Patient data were selected from an ongoing study (iBEAt [11]) on imaging biomarkers in diabetic kidney disease. The main inclusion criteria were type-2 diabetes, eGFR > 30 ml/min/1.73m^2^. All subjects signed informed consent prior to inclusion. For the purposes of this paper, the first 24 patients of one recruiting site were selected, excluding cases where DCE-MRI data were deemed to be of insufficient quality. Quality control consisted of visual inspecting of the raw images in a DICOM viewer. Any images that show artifacts, signal drop-outs, data truncation or extreme noise levels suggesting coil issues, were excluded.

Patients were scanned on a 3 T MR system (Magnetom Prisma, Siemens, Erlangen, Germany), feet first with an 18-channel phased array body coil. Apart from DCE-MRI, the scan protocol included a range of sequences including DIXON, T_1_/T_2_/T_2_*-mapping, diffusion weighted imaging and diffusion tensor imaging, ASL and phase contrast. DCE-MRI was performed in free breathing with a 2D fast gradient echo sequence with a non-selective saturation pulse prior to each slice readout. 2D acquisition was chosen because of the smaller acquisition time per slice as compared to a 3D volume acquisition. This renders the images less vulnerable to (respiratory) motion artifacts. Eight oblique slices through the kidney and one transverse slice through the descending aorta were acquired with acquired voxel size of 2.78 × 2.08 × 7.5 mm^3^ reconstructed to a voxel size of 1 × 1 × 7.5 mm^3^. The field of view was 400 × 400mm and a parallel imaging factor of 2 was employed, along with a partial Fourier factor of 7/8. Temporal resolution was 1.6s and total acquisition time was 7 min. Flip angle was 10 degrees, TR and TE were 179 ms and 0.97 ms, respectively, with an echo spacing of 2.2 ms. Image acquisition started 20s before bolus injection of 0.05 ml/kg gadoterate meglumine at a rate of 2 mL/s followed by a 20 mL saline flush at a rate of 2 mL/s.

DCE-MRI images were aligned slice-by-slice using model-driven registration [12]. Registration accuracy was evaluated by visual comparison of the coregistered dynamics against the original time series, and by comparing motion corrected versus uncorrected datasets. Motion correction results were rejected if the coregistration had created unrealistic deformations or had blurred out detail that was visible in the source images. Segmentation was performed on the registered data using k-means clustering [13]. Three clusters were created: one to represent the cortical voxels, one for inner medullary voxels and one for voxels containing partial voluming/outer medulla (on average 22% of all voxels labeled as kidney parenchyma). The latter were not used in further processing. Segmentation of the aorta for calculation of the arterial input function was performed by k-means clustering as well. Any voxels outside of the selected anatomical regions were manually removed. Average cortical, inner medullary, and aorta time–intensity curves were calculated from each data series.

### Implementation of the model

**Fig. 2:**
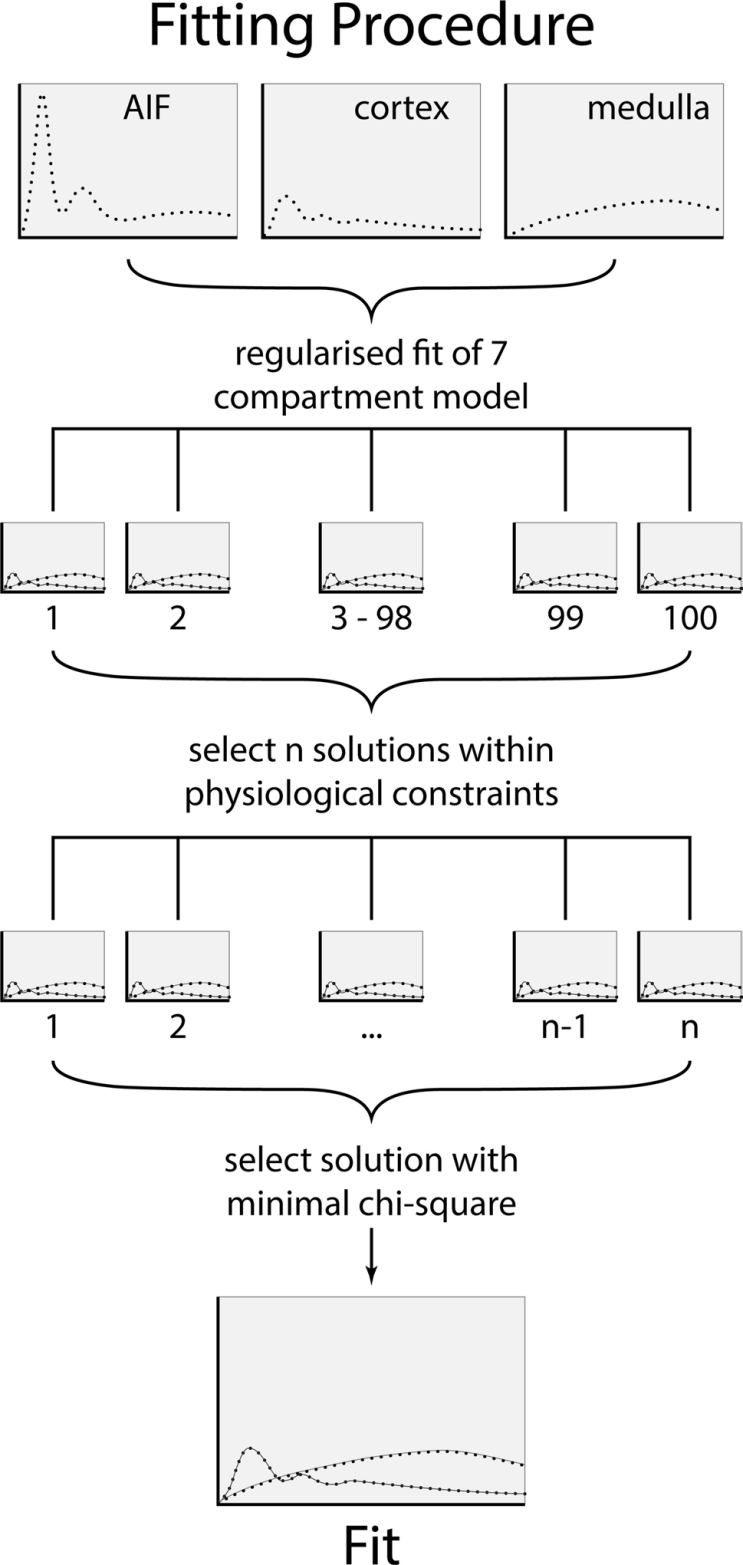
The fitting procedure depicted schematically. As part of the fitting procedure, a regularized unconstrained fit is performed 100 times with randomly chosen initial values. Next, the solutions within physiological bounds are selected. Last, the solution with the lowest chi-squared is selected

The model summarized in Eqs. 8 and 9 was implemented in Matlab (R2019a, MathWorks, Natick, MA, USA), using signals in arterial blood, cortex and inner medulla as input data and producing measurements of all 10 free parameters as outputs. Arterial and tissue concentrations were assumed to be proportional to the signal change with respect to the baseline level. A hematocrit of 0.45 was assumed to convert the aorta blood concentration to plasma concentration. For a given arterial plasma concentration, predicted tissue concentrations were calculated using Eqs. 8 and 9 with convolutions calculated as described in [14]. The model parameters were fitted to inner medullary and cortical signal curves simultaneously in a single regularized fit [15]. The model fit was repeated 100 times with different initial parameter values randomly chosen within physiological ranges, but without bounds on the fits. Of the 100 obtained solutions, only those solutions were selected with all parameters within physiological bounds. The solution with the lowest chi-square was selected as the result of the fit. The procedure is graphically depicted in Fig. 2.

### Simulated data collection

**Fig. 3:**
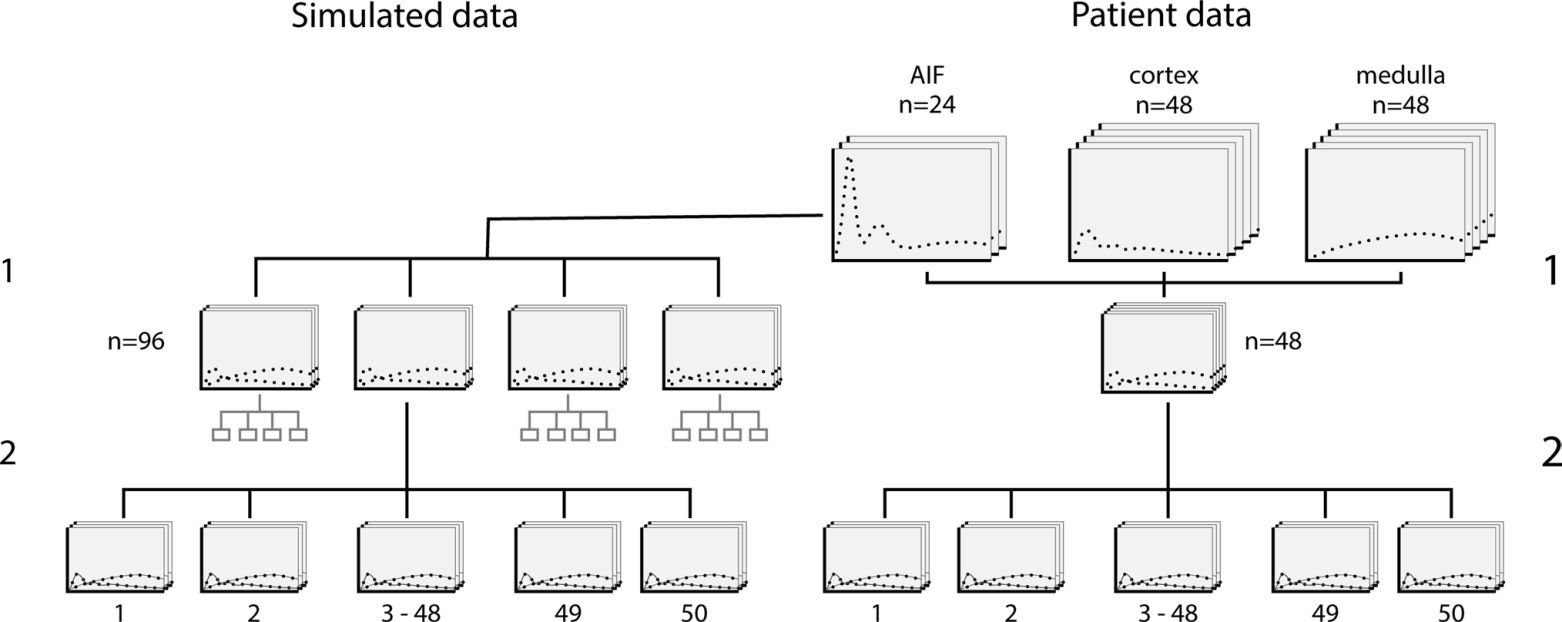
Data generation to determine stability and accuracy of the fitting procedure. Left: simulated data: (1) for each available arterial input function (aorta concentrations measured in patients) 4 sets of kidney tissue signal-time curves were generated, for a total of 96 unique curves with unique random choices of parameters, (2) the fitting procedure as depicted in Fig. 2 was applied 50 times to determine fitting stability and accuracy. Right: patient data: (1) for each patient, two datasets were available (left and right kidney), (2) the fitting procedure as depicted in Fig. 2 was applied 50 times to determine fitting stability

The aorta concentrations measured in the patients were used to generate kidney tissue signal–time curves with known ground-truth model parameters (Fig. 3). To determine realistic ground truth and bounds for all parameters, literature values were used where available. These were subsequently adjusted by data obtained by explorative model fits on patient data. Sets of ground truth values for all parameters were obtained from the normal distribution with a standard deviation of 20% around those theoretical values. Using those sets of ground truth values and the measured arterial plasma concentrations, signal–time curves were generated. Gaussian noise with a fixed standard deviation was added to the signals to obtain an arterial contrast-to-noise ratio (CNR) of 150, similar to the patient data. Arterial CNR was defined as the ratio between peak arterial signal change and baseline standard deviation.

### Data analysis

While the analysis is designed primarily to measure inner medullary perfusion, the 9 other parameters generated by the analysis are reported as well as they can be of use for additional quality control.

Patient data were used to verify that the model fitted the data well, compare results against known literature values, test sensitivity of the results to the choice of initial values, and compare shared parameters against a common reference model. Goodness of fit was assessed visually by inspection of fits overlaid on data. Results for all model parameters were reported as groupwise mean (standard deviation) and compared to literature values where available. In order to test model sensitivity to initial values, the fitting procedure was repeated 50 times for each dataset. This procedure is schematically depicted in Fig. 3. The coefficient of variation (CoV, defined as standard deviation/mean) was calculated over these 50 fits. If the iterated fit is perfectly stable, this CoV equals zero. Values for tubular flow F_T_ (GFR per unit of tissue volume) and F_cor_ were compared to values obtained using the current reference two-compartment filtration model [7]. The intraclass correlation coefficient ICC was used to measure agreement between both measurements (two-way mixed effects, consistency, single measurements).

The simulated data were used to determine sensitivity to initial values and accuracy of the model fit if used with noisy data. The bias with respect to the ground-truth value was used to determine accuracy of the model fit. To determine whether the model is sensitive enough to detect individual differences in F_med_, fitted F_med_ was compared to true F_med_ for all ground truth curves using the intraclass correlation coefficient (ICC, two-way mixed effects, absolute agreement, single measurement). Sensitivity of all parameters to initial values was measured in a similar way as in patient data by repeating the fitting procedure 50 times for each simulated dataset (Fig. 3) and quantify sensitivity using a CoV defined as standard deviation/mean.

## Results

### DCE data

**Fig. 4:**
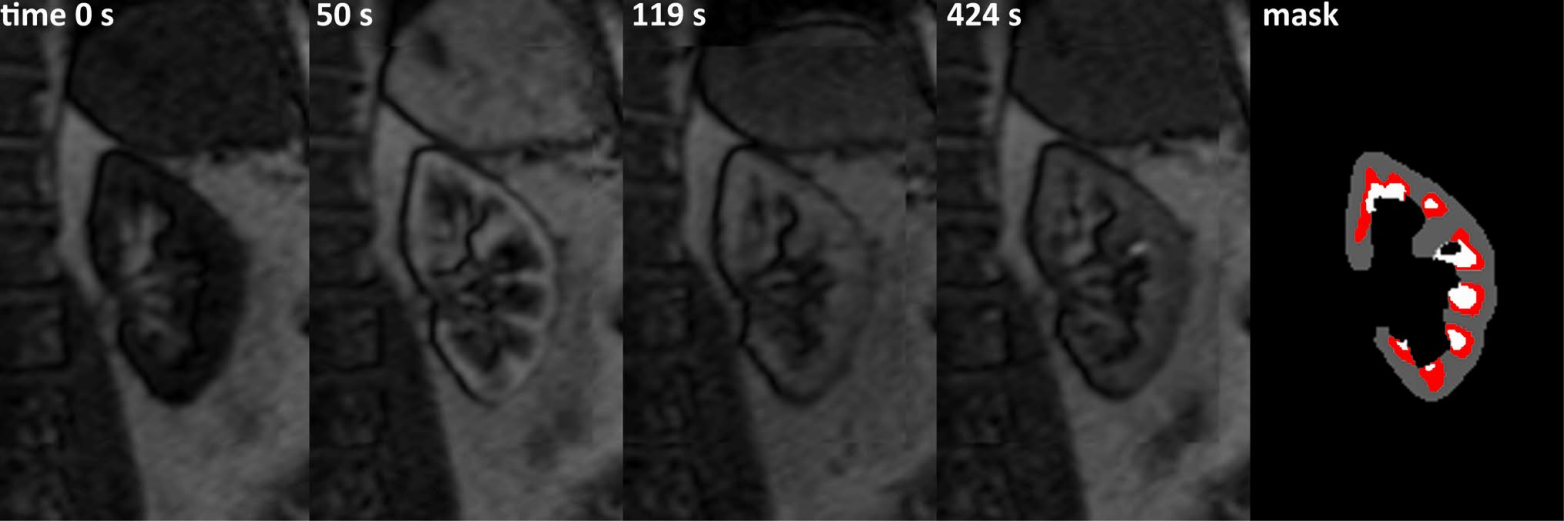
Representative DCE images obtained in a patient with diabetes. The *mask* image depicts the cluster results: the cortical cluster is depicted in gray, the inner medulla in white and the partial voluming/outer medulla is depicted in red since it is not used in the data analysis

Of the 24 DCE examinations available, four patients were excluded because of excessive inflow fluctuations in the AIF data (for details, see supplemental materials). An example of an AIF which was excluded alongside an included AIF is shown in the supplementary materials (Figure S1). In Fig. 4, a representative example of the pre-contrast and post-contrast DCE images in one patient is shown, alongside the segmentations.

### Measurement of inner medullary perfusion

**Fig. 5:**
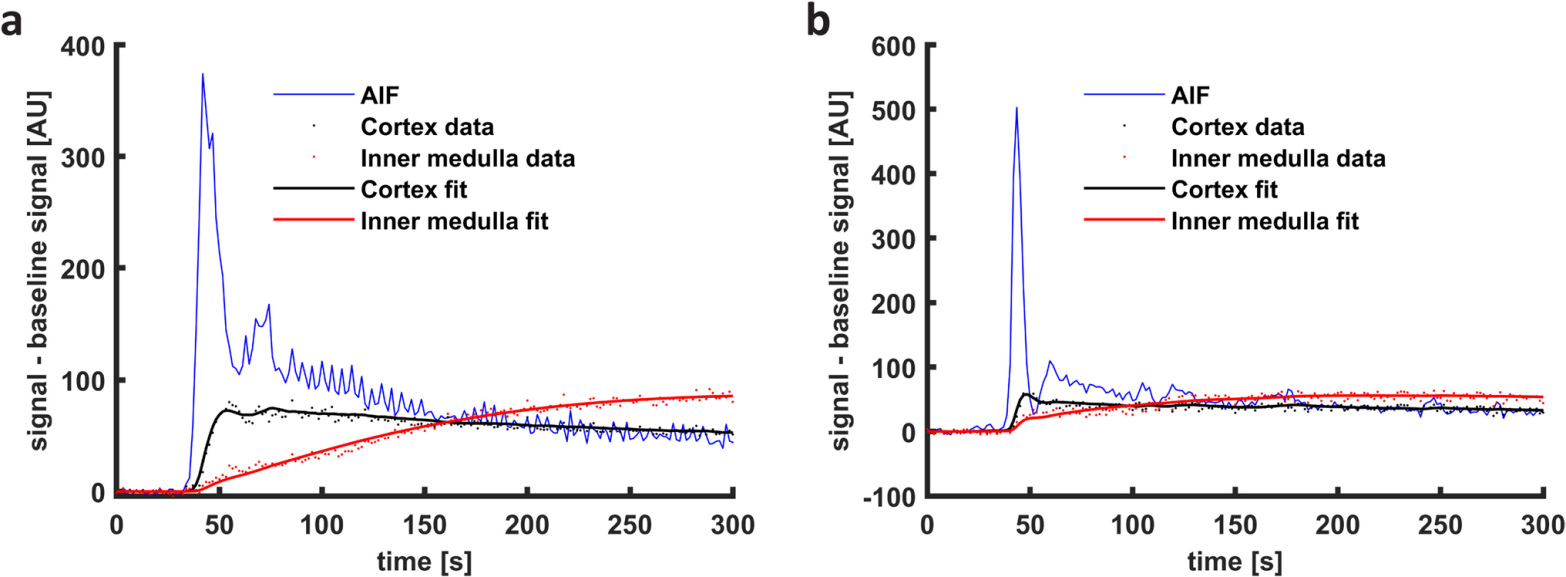
Model fits on real data. In general, capturing the first-pass peak remains challenging. In **a**, the first-pass peak is not very pronounced but yet is not completely captured by the model fit; while in **b** the first-pass peak of the inner medulla and to a lesser extent the cortex is not captured by the model fit. *AIF *arterial input function

**Fig. 6:**
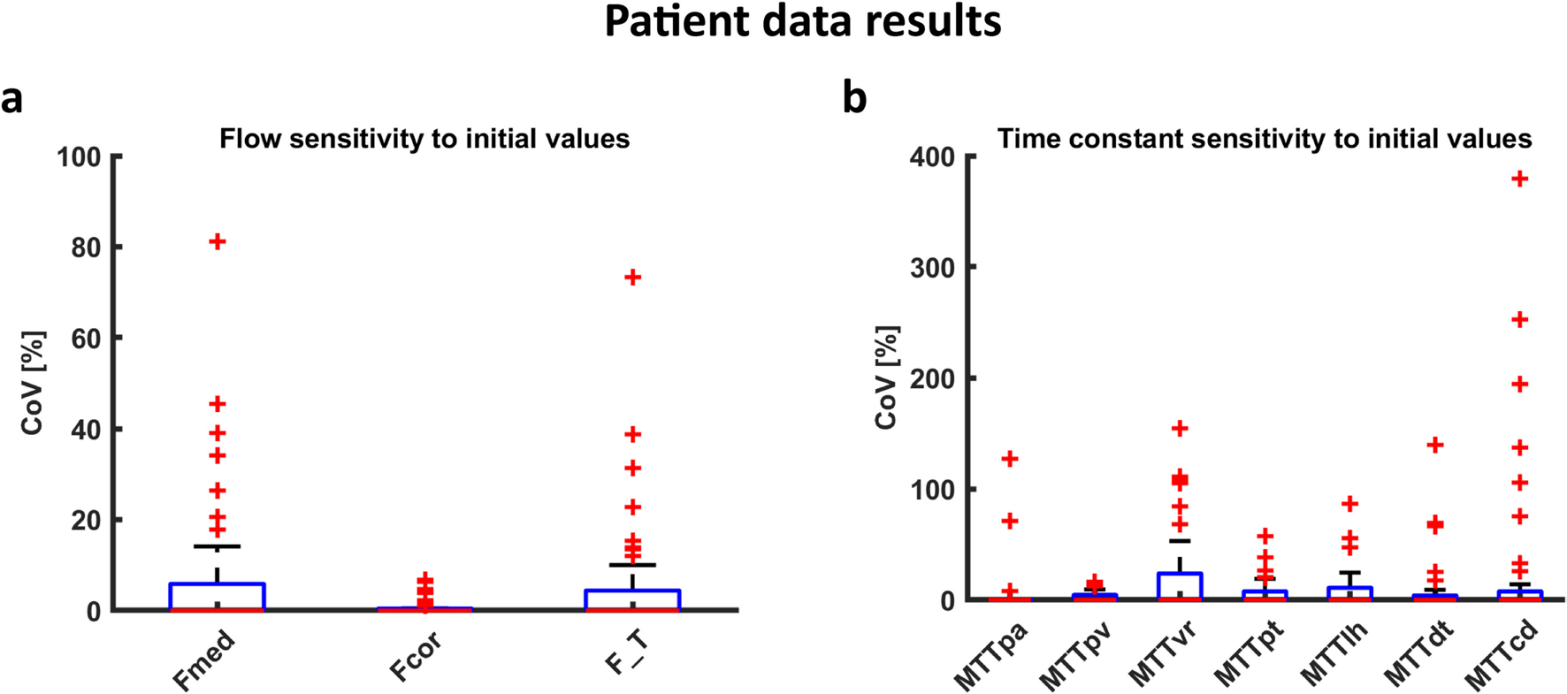
Patient data results. Sensitivity to initial values in terms of CoV of the seven-compartment model with the iterated fitting approach, based on 50 iterated fits of each of the 40 time–intensity curves; **a** CoV of inner medullary (F_med_) and cortical (F_cor_) perfusion and F_T_; **b** CoV of mean transit times for each compartment. Note the difference in scale of the y-axis. For all parameters, ideally the boxplots would lie invisibly on the x-axis because this indicates perfect stability of the fits. However, of interest are the outliers (red crosses), which reflect unstable fits despite the iterative fitting approach. In contrast, the proximity of the boxes to the x-axis reflect the excellent fitting stability in most curves, resulting in a CoV close to zero when the iterated fitting procedure is repeated 50 times. *F*_*med*_ inner medullary perfusion, *F*_*cor*_ cortical perfusion, *T*_*pa*_ arterial plasma compartment, *T*_*pv*_ venous plasma compartment, *T*_*vr*_ vasa recta compartment, *T*_*pt*_ proximal tubules compartment, *T*_*lh*_ loop of Henle compartment, *T*_*cd*_ collecting duct compartment

**Fig. 7:**
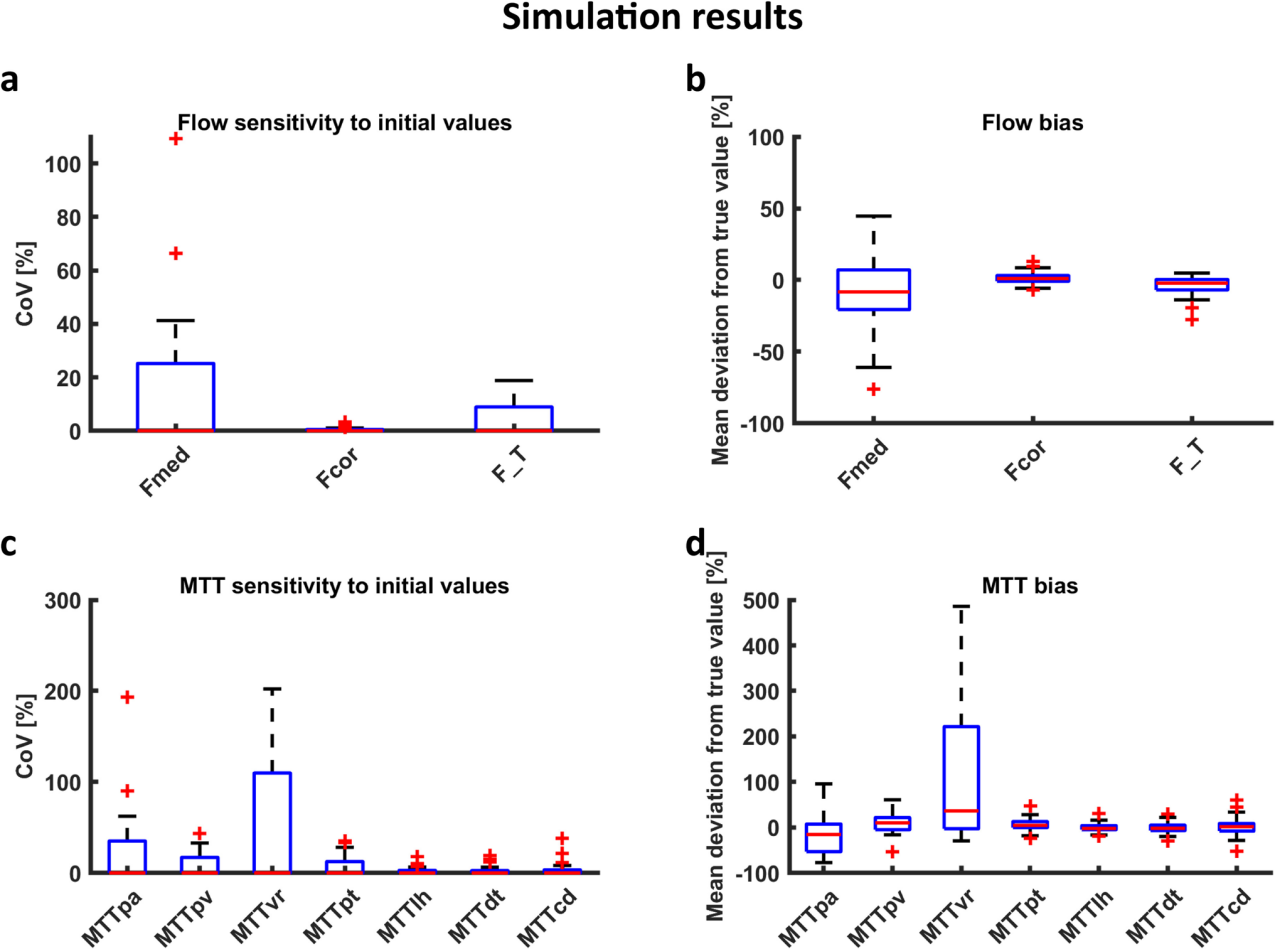
Simulation results. Sensitivity to initial values: **a** CoV and **b** bias of the flows and **c** CoV and **d** bias of the mean transit times measured using the seven-compartment model with the iterated fitting approach, based on 50 iterated fits of each of the 40 ground truth curves. As in Fig. 6, the outliers (red crosses) are of main interest since they reflect unstable fits despite the iterative fitting approach. *F*_*med*_ inner medullary perfusion, *F*_*cor*_ cortical perfusion, *MTT*_*pa*_ arterial plasma compartment, *MTT*_*pv*_ venous plasma compartment, *MTT*_*vr*_ vasa recta compartment, *MTT*_*pt*_ proximal tubules compartment, *MTT*_*lh*_ loop of Henle compartment, *MTT*_*cd*_ collecting duct compartment

In Fig. 5, fits of the seven compartment model to time–intensity curves obtained in patients are shown. A small mismatch in the first pass was often observed, but overall, the model fits the data well. In the supplementary materials, the root-mean-square errors of the fits of the patient curves are provided. Table 2 summarizes the key numerical results in patients and simulated data, and Figs. 6 and 7 show more detailed data distributions for patient data and simulations, respectively. Average (stdev) inner medullary perfusion was 37 (23) mL/100 mL/min. Sensitivity to initial values as reflected by the CoV between iterated fits was median 0.0 (0.0–5.8) %. However, there were outliers with CoVs up to 81%. In the simulations, noise caused a systematic underestimation of inner medullary perfusion by around 8% compared to ground truth. The CoV measuring the sensitivity to initial values was median 0.0 (0.0–25) % in simulations, with outliers up to ~ 80%. A CoV of 0.0 indicates good stability of the fit Table 3.

**Table 2:** Parameter values, fitting stability and accuracy (deviation from true value in simulations) as obtained by fitting of the 7-compartment model on both patient data and simulated data

	Patient data		Simulations		
	Fitted value	Sensitivity to initial value (CoV)	Fitted value	Deviation from true value	Sensitivity to initial value (CoV)
	Groupwise mean (SD)	Groupwise median % (IQR)	Groupwise mean (SD)	Groupwise median % (IQR)	Groupwise median % (IQR)
F_med_* (mL/100 mL/min)	37 (23)	0.0 (0.0–5.8)	70 (31)	− 8.3 (− 21–7.0)	0.0 (0.0–25)
F_cor_ (mL/100 mL/min)	246 (73)	0.0 (0.0–0.4)	249 (52)	1.0 (-0.9–3.1)	0.0 (0.0–0.47)
F_T_† (mL/100 mL/min)	55 (21)	0.0 (0.0–4.4)	72 (23)	− 2.2 (− 6.9–0.4)	0.0 (0.0–8.9)
MTT_PA_ (s)	1.3 (1.8)	0.0 (0.0–0.0)	1.5 (0.9)	− 16 (− 53–7.3)	0.0 (0.0–35)
MTT_PV_ (s)	11 (3.9)	0.0 (0.0–4.5)	7.5 (2.3)	10 (-5.1–21)	0.0 (0.0–17)
MTT_VR_ (s)	140 (113)	0.1 (0.0–24)	32 (41)	36 (− 2.9–221)	0.0 (0.0–110)
MTT_PT_ (s)	52 (19)	0.0 (0.0–7.6)	43 (9.1)	4.5 (− 0.4–13)	0.0 (0.0–12)
MTT_LH_ (s)	80 (70)	0.0 (0.0–11)	77 (14)	− 2.4 (− 5.0–3.9)	0.0 (0.0–2.8)
MTT_DT_ (s)	36 (53)	0.0 (0.0–4.0)	29 (7.1)	− 1.8 (− 7.0–4.8)	0.0 (0.0–2.5)
MTT_CD_ (s)	30 (21)	0.0 (0.0–7.6)	41 (9.9)	1.5 (− 8.2–8.6)	0.0 (0.0–3.4)

*Derived from F_cor_ and E_med_ by E_med_(1-E_FF_)F_cor_; † Derived from F_cor_ by multiplication by E_FF_

*CoV* coefficient of variation, *F*_*med*_ medullary perfusion, *F*_*cor*_ cortical perfusion, *T*_*PA*_ arterial plasma compartment, *F*_*T*_ tubular flow, *T*_*PV*_ venous plasma compartment, *T*_*VR*_ vasa recta compartment, *T*_*PT*_ proximal tubules compartment, *T*_*LH*_ loop of Henle compartment, *T*_*CD*_ collecting duct compartment, *E*_*med*_ medullary extraction fraction, *E*_*FF*_ filtration fraction

**Table 3:** Groupwise averages of parameter estimations for the seven compartment alongside estimates of the two-compartment model

		Seven-compartment model		Two-compartment model	
Parameter	Unit	Mean	SD	Mean	SD
F_cor_	mL/100 mL/min	246	73	177	52
T_PA_	S	1.3	1.8	11.1	5.5
F_T_	mL/100 mL/min	55	21	49	18

*F*_*cor*_ cortical perfusion, *T*_*PA*_ arterial plasma compartment transit time, *F*_*T*_ tubular flow

**Fig. 8:**
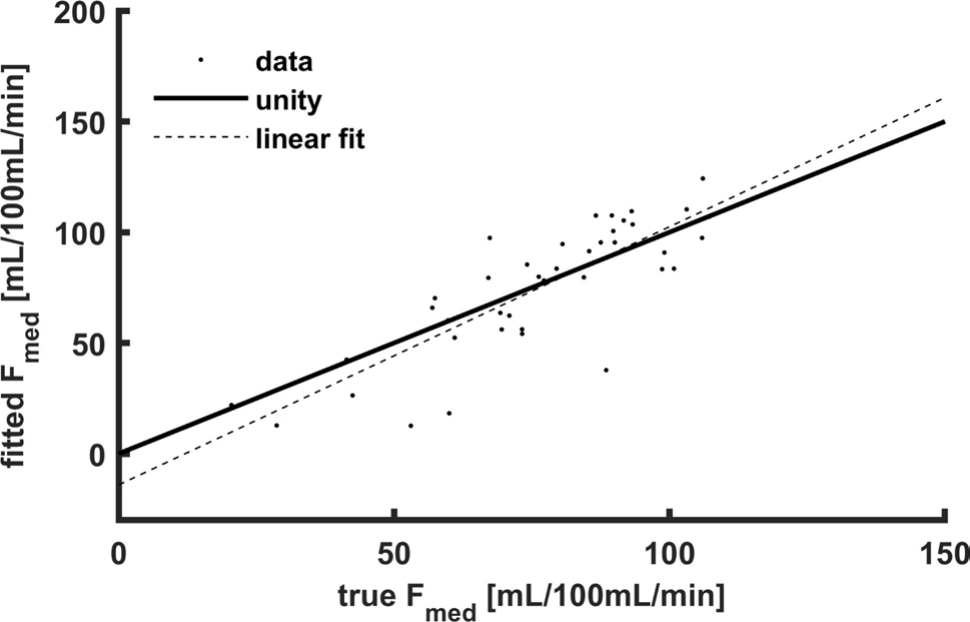
Fitted inner medullary perfusion F_med_ versus true F_med_ for the iterated fitting approach. The slope of the regression line is 1.2 with an *R*^2^ of 0.67 (*p* < 10^−10^). *F*_*med*_ inner medullary perfusion

Figure 8 shows the estimated inner medullary perfusion against ground truth in all simulations. The agreement was reasonable as shown by the regression line (slope 1.2), but the correlations were relatively poor (*R*^2^ = 0.67) and variable (ICC = 0.77).

### Measurement of secondary parameters

Measurement of cortical perfusion was reasonably accurate with a bias of + 1.0 (− 0.9–3.1) % compared to ground truth in simulations and sensitivity to initial values was low with the 75th percentile of CoV below 0.5% in both simulations and patient data. Average cortical plasma flow obtained in patients was 246 ml/100ml/min which is at the high end of literature values, similar to a reference measurement with H2O-labeled PET (258 mL/100mL/min, assuming Hct of 0.45). F_T_ could be measured reasonably accurately with a bias of − 2.2 (− 6.9–0.4) % and reasonably precise with the 75th percentile of CoV below 9% in both simulations and patient data.

Estimates of the MTT of tubular compartments were relatively accurate (bias from 1.5% to 4.5%), but the MTTs of the vascular compartments were more biased (bias from 10 to 36%). Sensitivity to initial values of the MTTs was reasonable for the tubular compartments (75th percentile of the CoV up to 12%). However, this sensitivity was markedly increased especially for MTT_VR_, with the 75th percentile of the CoV equaling 110%.

**Fig. 9:**
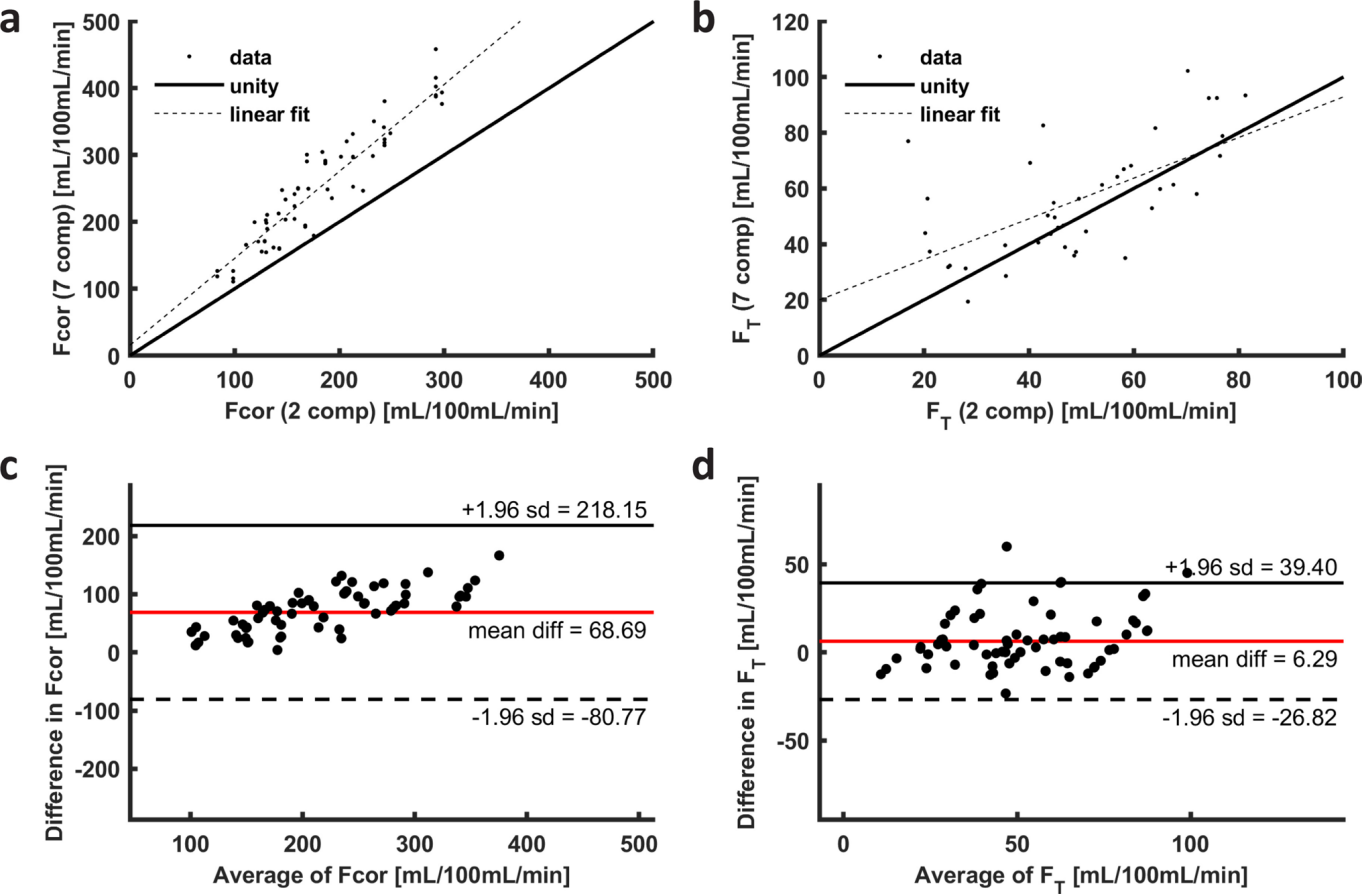
a A comparison of cortical perfusion F_cor_ as measured by the two-compartment model and the seven-compartment model. Cortical perfusion is higher by around 40% by the 7-compartment model compared to the 2-compartment model. The linear fit corresponds to F_cor,7cmp_ = 18 + 1.3F_cor,2cmp_ (*p* < 10^−16^) and R^2^ equals 0.84; **b** the same for F_T_. F_T_ is higher by around 10% by the 7-compartment model compared to the 2-compartment approach. The linear fit corresponds to F_T,7cmp_ = 20 + 0.73 F_T,2cmp_ (*p* < 10^−5^). *R*^2^ equals 0.39. The comparison is carried out on patient data**; c** Bland–Altman plot of the difference between 2- and 7-compartment models. The overestimation of cortical perfusion by the 7-compartment model compared to the 2-compartment model seems to increase with higher perfusion; **d** Bland–Altman plot of F_T_ estimated by the 2- and 7-compartment models. *F*_*cor,7cmp*_ cortical perfusion obtained from 7-compartment model, *F*_*cor,2cmp*_ cortical perfusion obtained from 2-compartment model, *F*_*T,7cmp*_ glomerular filtration rate obtained from 7-compartment model, *F*_*T,2cmp*_ tubular flow obtained from 2-compartment model

Correlation to parameter estimates obtained using the 2-compartment filtration model is shown in Fig. 9. Compared to the 2-compartment filtration model, the 7-compartment model produces higher cortical perfusion. The ICC for cortical perfusion obtained with the two- and seven-compartment models was 0.87 and for F_T_ the ICC was 0.63.

## Discussion

We present a pharmacokinetic model for contrast passage through the kidneys, designed for measurement of inner medullary perfusion. Compared to models published earlier, the model follows renal physiology more closely and separates cortex and medulla [5–9]. As a consequence, ten free parameters were used, rendering the results vulnerable for overfitting. However, with the suggested iterative and regularized fitting approach, we show that relatively stable results can be obtained even for renal inner medullary perfusion. Compared to the model proposed by Lee et al. [9], we enable direct measurement of cortical and inner medullary perfusion.

### Parameter estimations

The main parameter of interest for this project is inner medullary perfusion. In simulations, a comparison between true inner medullary perfusion versus the perfusion obtained from the model fit, yielded a reasonable correlation (ICC 0.77) and a 1.2 slope of the linear fit. This indicates that the model is sensitive to changes in inner medullary perfusion, which is of interest for pre–post-experiments, where subjects act as their own control. Values for inner medullary perfusion as measured by this model were higher compared to values reported earlier from ASL measurements [16]. As mentioned in the introduction, the ASL tracer is largely decayed before it reaches the renal medulla and (inner) medullary perfusion might be underestimated. This might partly be overcome using PASL since labeling is performed closer to the kidney. In contrast, we report relatively low inner medullary perfusion in comparison to measurements obtained by ^15^O-labeled water PET in obese subjects [17]. However, these values were derived from a model which only considers the kidney as a whole and does not discriminate between cortex and medulla [18]. Our simulations indicated an underestimation of around 8% for inner medullary flow, so a certain degree of underestimation is likely.

Cortical perfusion and F_T_ could be compared directly to the more conventional two-parameter model as proposed initially by [5]. As visible in Fig. 9, cortical perfusion as measured by the 7-compartment model was systematically higher compared to the value obtained from the 2-compartment model. However, the 2-compartment model was applied to the parenchymal signal enhancement curves, so this “cortical” perfusion and F_T_ actually represent perfusion and F_T_ of the entire kidney. Therefore, this likely reflects an actual difference rather than an overestimation. However, as illustrated by an ICC of 0.87 for cortical perfusion, consistency between models was excellent. For F_T_ measurement, the ICC of 0.63 indicated moderate consistency though the systematic difference was much smaller.

### Model assumptions

The model presented here builds on the multicompartment modeling approach for cortex and medulla as presented by Lee et al. [9]. However, since that model used a single arterial compartment not separated in arterioles and vasa recta, it is not able to provide estimates of medullary perfusion. Furthermore, the initial 7-compartment model was simplified to a 3-compartment model for actual implementations.

We assumed a linear relation between signal intensity and contrast agent concentration. Only a quarter dose of contrast agent was administered to avoid high concentrations during first pass, and for low concentrations, this assumption is reasonable. Next, the compartmental approach assumes fast exchange of water in renal cortical and medullary tissue and consequently a mono-exponential signal decay, which might not hold in renal tubules and vessels [19]. A fixed value for blood hematocrit was assumed in all vessels for conversion of blood concentrations to plasma and vice versa. Small vessel hematocrit however is assumed to be significantly lower [6]. Last, contrast agent absorption and secretion were neglected in derivation of this model since gadoterate meglumine is predominantly passively excreted via glomerular filtration [9].

### Cortex and inner medulla segmentation

Segmentation of cortex and inner medulla was performed using k-means clustering as proposed by [20], based on the inherently different signal enhancement curves of cortex and medulla. The three clusters generated for each kidney were appointed to cortex, inner medulla and a region of partial volume or potentially outer medulla. The latter cluster was excluded. However, if it is assumed to represent partial volume, which seems reasonable with a reconstructed voxel size of 1 × 1x7.5mm^3^, it can be fitted along with the cortical and medullary curves by adding a single extra parameter defining the fractional medullary contribution to the curve.

### Model fit stability and accuracy

The model fit in general was reasonably well, but the model fit systematically underestimated the height of the first-pass peak. In line with other models, we used an exponential vascular input response function which might not capture the complexity of renal arterial vasculature. The complexity of the model (ten free parameters) creates a risk of overfitting. Note however that these 10 free parameters are obtained from two distinct curves, the cortical and inner medullary curve. For some, but not all signal enhancement curves, the model fit ended up in local minima. This was partly tackled by the iterative fitting approach with varying initial values, which is more likely to identify the global optimum. Furthermore, we used a regularized fitting approach [15]. This resulted in a median coefficient of variation below 10% for all parameters of interest, but there were outliers up to a CoV of around 80% for F_med_. Note however that this reflects the relative difference of a very small quantity, which in absolute numbers is only around 10–30 ml/min/100ml.

Measurements of the mean transit times were inaccurate, and potentially this reflects some degree of overparameterization in the modeling. Potentially the bolus dispersion in some spaces can be safely ignored, which might reduce the number of free parameters and can potentially improve stability of the fit. Test runs showed that fixing of MTT_LH_ might improve stability without affecting of F_cor_, F_med_, F_T_ estimation too much.

## Conclusion

In conclusion, we present a multicompartment model which reflects renal physiology and enables separate measurement of cortical and medullary perfusion, along with tubular flow (GFR per unit of kidney volume). The model was applied to MR renography data obtained in diabetic subjects. Since in vivo measurement of human medullary perfusion up to now is impossible, no validation to a reference standard could be performed. Further studies should show whether the model is able to detect intervention induced changes in medullary perfusion.

## Supplementary Information

Below is the link to the electronic supplementary material.

Supplementary file1 (DOCX 120 KB)

## Data Availability

Not applicable.
