# Supplementary material for: A method to measure renal inner medullary perfusion using MR renography
Source: MAGMA. 2025 Feb 15;38(5):791–802. doi: 10.1007/s10334-025-01225-7 (PMC12497669; doi:10.1007/s10334-025-01225-7)
Supplement: Supplementary file 1 — Supplementary file1 (DOCX 120 KB) [file 10334_2025_1225_MOESM1_ESM.docx]

# Supplementary materials

## Theory

For each compartment, a differential equation and a conservation of mass equation can be derived. The differentials equations for each compartment are:

$$V_{PA}\frac{dC_{PA}}{dt}=F_{cor}C_{A}-\left( F_{T}+F_{med}+F_{PV,PA} \right)C_{PA} (1)$$

$$V_{VR}\frac{dC_{VR}}{dt}=F_{med}C_{PA}-F_{PV,VR}C_{VR} (2)$$

$$V_{PV}\frac{dC_{PV}}{dt}=F_{PV,P}C_{PA}+F_{PV,VR}C_{VR}-F_{O,PV}C_{PV} (3)$$

$$V_{PT}\frac{dC_{PT}}{dt}=F_{PT,PA}C_{PA}-F_{LH,PT}C_{PT} (4)$$

$$V_{LH}\frac{dC_{LH}}{dt}=F_{LH,PT}C_{PT}-F_{DT,LH}C_{LH} (5)$$

$$V_{DT}\frac{dC_{DT}}{dt}=F_{DT,LH}C_{LH}-F_{CD,DT}C_{DT} (6)$$

$$V_{CD}\frac{dC_{CD}}{dt}=F_{CD,DT}C_{DT}-F_{O,CD}C_{CD} (7)$$

The conservation of mass equations are:

$$F_{cor}=F_{PV,PA}+F_{med}+F_{T}$$

$$F_{med}+\left( f_{CD}+f_{LH} \right)F_{T}=F_{PV,VR}$$

$$F_{PV,PA}+F_{PV,VR}+\left( f_{PT}+f_{DT} \right)F_{T}=F_{O,PV}$$

$$F_{T}=f_{PT}F_{T}+F_{LH,PT}$$

$$F_{LH,PT}=f_{LH}F_{T}+F_{DT,LH}$$

$$F_{DT,LH}=f_{DT}F_{T}+F_{CD,DT}$$

$$F_{CD,DT}=f_{CD}F_{T}+F_{O,CD}$$

However, since the resorption fractions f_PT_, f_LH_, f_DT_ and f_CD_ do not show up in the differential equations and the flows F_LH,PT_, F_DT,LH_, F_CD,DT_ and F_O,CD_ cancel out, apart from the first one these equations are neither useful for simplification of the model nor for elimination of parameters.

Now we define $H_{X}=1/{MTT_{X}}e^{-\frac{t}{MTT_{X}}}$ (propagator through compartment X) and ${MTT}_{X}={V_{X}}/{F_{out}}$ (time constant of X). The solutions of the differential equations are of the form:

$$C_{X}\left( t \right)=\frac{F_{in}}{F_{out}} H_{X}*C_{in}\left( t \right)$$

This leads to:

$${V_{PA}C}_{PA}\left( t \right)=\frac{1}{F_{T}+F_{med}+F_{PV,PA}}H_{PA}*{F_{cor}C}_{A}\left( t \right)=H_{PA}*C_{A}\left( t \right)$$

$$V_{VR}C_{VR}\left( t \right)=\frac{F_{med}}{F_{PV,VR}}H_{VR}*C_{PA}\left( t \right)$$

$$V_{PV}C_{PV}\left( t \right)=\frac{F_{PV,PA}}{F_{O,PV}}H_{PV}*C_{PA}\left( t \right)+\frac{F_{med}}{F_{O,PV}}H_{PV}*H_{VR}*C_{PA}(t)$$

$$V_{PT}C_{PT}(t)=\frac{F_{T}}{F_{LH,PT}} H_{PT}*C_{PA}(t)$$

$$V_{LH}C_{LH}(t)=\frac{F_{LH,PT}}{F_{DT,LH}} H_{LH}*C_{PT}(t)$$

$$V_{DT}C_{DT}\left( t \right)=\frac{F_{DT,LH}}{F_{CD,DT}}H_{DT}*C_{LH}(t)$$

$$V_{CD}C_{CD}\left( t \right)=\frac{F_{CD,DT}}{F_{O,CD}}H_{CD}*C_{DT}(t)$$

Next, we define the residue function $R_{X}={MTT}_{X}H_{X}=e^{-\frac{t}{{MTT}_{X}}}$ and we introduce an extraction fraction for each pair of compartments X and Y $E_{YX}$ from X to Y:

$$E_{Y,X}=\frac{F_{Y,X}}{\sum_{Y} F_{Y,X}}$$

This equals 1 for each compartment except the plasma (P) compartment. This gives:

$${V_{PA}C}_{PA}\left( t \right)={MTT}_{PA}H_{PA}*{F_{cor}C}_{A}\left( t \right)=R_{PA}*{F_{cor}C}_{A}\left( t \right)$$

This equals equation 1 from the main manuscript.

$$V_{PV}C_{PV}\left( t \right)={F_{PV,PA}MTT}_{PV}H_{PV}*C_{PA}\left( t \right)+{F_{med}MTT}_{PV}H_{PV}*H_{VR}*C_{PA}\left( t \right)$$

$$={F_{PV,PA}R}_{PV}*H_{PA}*C_{A}\left( t \right)+{F_{med}R}_{PV}*H_{VR}*H_{PA}*C_{A}\left( t \right)$$

$$=R_{PV}*E_{PV,PA}H_{PA}*F_{cor}C_{A}\left( t \right)+R_{PV}*H_{VR}*E_{VR,PA}H_{PA}*F_{cor}C_{A}\left( t \right)$$

$$V_{VR}C_{VR}\left( t \right)=F_{med}{MTT}_{VR}H_{VR}*C_{PA}\left( t \right)=R_{VR}*{E_{VR,PA}H}_{PA}*{F_{cor}C}_{A}\left( t \right)$$

Now define:

$$E_{med}=\frac{E_{VR,PA}}{\left( 1-E_{FF} \right)}$$

From (which follows from the conservation of mass)l:

$$E_{FF}+E_{PV,PA}+E_{VR,PA}=1$$

E_PV,PA_ can be derived:

$$E_{PV,PA}=\left( 1-E_{med} \right)(1-E_{FF})$$

Substitute in the equations for V_PV_C_PV_ and V_VR_C_VR_:

$$V_{PV}C_{PV}\left( t \right)={E_{PV,PA}MTT}_{PV}H_{PV}*\left( 1-E_{med} \right)(1-E_{FF})H_{PA}*F_{cor}C_{A}\left( t \right)+{MTT}_{PV}H_{PV}*H_{VR}*E_{med}(1-E_{FF})H_{PA}*F_{cor}C_{A}\left( t \right)$$

$$V_{VR}C_{VR}\left( t \right)=R_{VR}*{E_{med}(1-E_{FF})H}_{PA}*{F_{cor}C}_{A}\left( t \right)$$

Which equal equations 2 and 3 from the main manuscript. Continue with the tubular equations (equation 4-7 in the main manuscript):

$$V_{PT}C_{PT}\left( t \right)=F_{T}{MTT}_{PT}H_{PT}*C_{PA}\left( t \right)=R_{PT}*{E_{FF}H}_{PA}*{F_{cor}C}_{A}\left( t \right)$$

$$V_{LH}C_{LH}\left( t \right)=F_{LH,PT}{{MTT}_{LH}H}_{LH}*C_{PT}\left( t \right)=F_{LH,PT}R_{LH}*\frac{F_{T}}{F_{LH,PT}}H_{PT}*H_{PA}*C_{A}\left( t \right)$$

$$=F_{T}R_{LH}*H_{PT}*H_{PA}*C_{A}\left( t \right)=R_{LH}*H_{PT}*{E_{FF}H}_{PA}*{F_{cor}C}_{A}\left( t \right)$$

$$V_{DT}C_{DT}\left( t \right)=F_{DT,LH}T_{DT}H_{DT}*C_{LH}\left( t \right)=F_{DT,LH}R_{DT}*\frac{F_{LH,PT}}{F_{DT,LH}}H_{LH}*\frac{F_{T}}{F_{LH,PT}}H_{PT}*H_{PA}*C_{A}\left( t \right)$$

$$=F_{T}R_{DT}*H_{LH}*H_{PT}*H_{PA}*C_{A}\left( t \right)=R_{DT}*H_{LH}*H_{PT}*E_{FF}H_{PA}*F_{cor}C_{A}\left( t \right)$$

$$V_{CD}C_{CD}\left( t \right)=F_{CD,DT}{MTT}_{CD}H_{CD}*C_{DT}\left( t \right)=F_{CD,DT}R_{CD}*\frac{F_{DT,LH}}{F_{CD,DT}}H_{DT}*\frac{F_{LH,PT}}{F_{DT,LH}}H_{LH}*\frac{F_{T}}{F_{LH,PT}}H_{PT}*H_{PA}*C_{A}\left( t \right)=F_{T}R_{CD}*H_{DT}*H_{LH}* H_{PT}*H_{PA}*C_{A}\left( t \right)=R_{CD}*H_{DT}*H_{LH}* H_{PT}*E_{FF}H_{PA}{F_{cor}C}_{A}(t)$$

## Exclusion of AIFs

Four AIFs were excluded from analysis because of excessive inflow artifacts. The ratio of the interquartile range divided by the median value of the tail of the curve (from t>60, excluding the first-pass peak) was calculated. The curves with a ratio>1 were selected and visually assessed to confirm excessive inflow artifacts. These curves were excluded. For an example, see figure S1.

# Supplementary tables

Table S1 Root-mean-squared error of each fit, normalized to median signal strength.

|  |  | RMS Error |
| --- | --- | --- |
| Patient 1 | Left | 0.0594 |
|  | Right | 0.0636 |
| Patient 2 | Left | 0.0867 |
|  | Right | 0.0797 |
| Patient 3 | Left | 0.0934 |
|  | Right | 0.0869 |
| Patient 4 | Left | 0.0857 |
|  | Right | 0.0659 |
| Patient 5 | Left | 0.0864 |
|  | Right | 0.0775 |
| Patient 6 | Left | 0.0806 |
|  | Right | 0.0768 |
| Patient 7 | Left | 0.0871 |
|  | Right | 0.0728 |
| Patient 8 | Left | 0.0917 |
|  | Right | 0.0651 |
| Patient 9 | Left | 0.0829 |
|  | Right | 0.0628 |
| Patient 10 | Left | 0.1109 |
|  | Right | 0.0941 |
| Patient 11 | Left | 0.1239 |
|  | Right | 0.0693 |
| Patient 12 | Left | 0.0822 |
|  | Right | 0.0745 |
| Patient 13 | Left | 0.0641 |
|  | Right | 0.0653 |
| Patient 14 | Left | 0.1180 |
|  | Right | 0.0824 |
| Patient 15 | Left | 0.1243 |
|  | Right | 0.0759 |
| Patient 16 | Left | 0.0832 |
|  | Right | 0.0717 |
| Patient 17 | Left | 0.1216 |
|  | Right | 0.1028 |
| Patient 18 | Left | 0.1087 |
|  | Right | 0.0813 |
| Patient 19 | Left | 0.0698 |
|  | Right | 0.0803 |
| Patient 20 | Left | 0.0550 |
|  | Right | 0.0555 |

## Supplementary figures

**
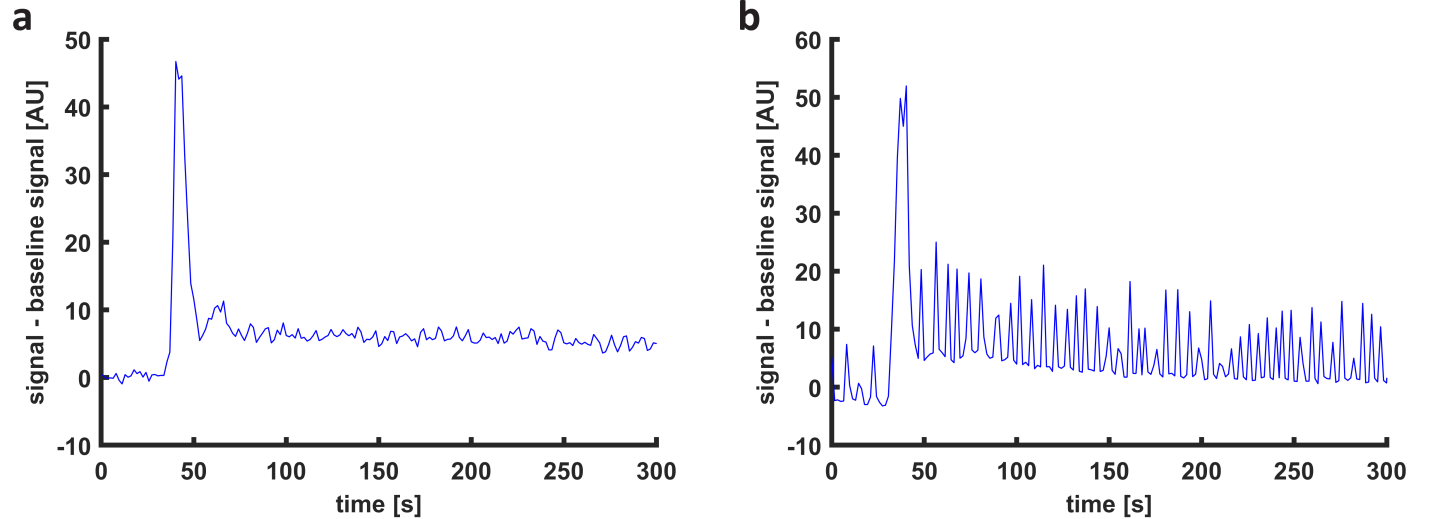
Figure S1:** good quality AIF **(a)** alongside an AIF which was excluded because of excessive noise **(b)**

*AIF: arterial input function*
